# Effects of prefermentative cold soak on polyphenols and volatiles of Aglianico, Primitivo and Nero di Troia red wines

**DOI:** 10.1002/fsn3.817

**Published:** 2019-01-30

**Authors:** Giuseppe Gambacorta, Antonio Trani, Cristina Fasciano, Vito Michele Paradiso, Michele Faccia

**Affiliations:** ^1^ Department of Soil, Plant and Food Science University of Bari Aldo Moro Bari Italy; ^2^ CIHEAM‐IAMB ‐ International Centre for Advanced Mediterranean Agronomic Studies Valenzano (BA) Italy

**Keywords:** Aglianico, cold soak, Nero di Troia, polyphenols, Primitivo, volatiles

## Abstract

Effectiveness of prefermentative cold soak (PCS) on polyphenols and volatiles extraction during winemaking of three red grape cultivars grown in southern Italy (Aglianico, Primitivo and Nero di Troia) was investigated. Four 200‐L stainless steel horizontal rotary wine fermenters were used. The main result was that PCS improved the extraction of polyphenols and increased the antioxidant activity of wines. Extraction of proanthocyanidins was enhanced (+25%, +14% and +7% for Aglianico, Primitivo and Nero di Troia, respectively) and, consequently, the ratio flavans reactive with vanillin/proanthocyanidin was reduced, thus potentially favoring the chromatic and tannic stabilization of wines. As regards volatiles, PCS increased ester compounds at levels above their odor thresholds, potentially conferring fruity odor to wines. In conclusion, PCS could be favorably introduced in winemaking to enhance the enological potential of Aglianico, Primitivo and Nero di Troia wines.

## 1 INTRODUCTION

Red wine quality is strongly affected by the phenolic and volatile content and composition, which are responsible for sensory characteristics such as color, texture, taste, and aroma. Phenols and volatiles in red wine depend on the grape composition, winemaking technology, and storage conditions (Gambuti, Capuano, Lisanti, & Moio, 2010; Piñeiro et al., 2006).

Phenol compounds play a crucial role both in plant physiology and in human health. They are plant secondary metabolites and are involved in the responses against biotic and abiotic stress (Winkel‐Shirley, 2002). Some phenolic compounds show biological activity, including antibacterial, anticarcinogenic, anti‐inflammatory, and cardio‐protective properties, attributed to their antioxidant and antiradical activity (Fresco, Borges, Diniz, & Marques, 2006; King, Bomser, & Min, 2006; Santos‐Buelga & Scalbert, 2000; Soleas, Grass, Josephy, Goldberg, & Diamantis, 2006). Phenolic compounds are located in skins, pulp, and seeds of grapes and are partially extracted during winemaking. Flavonoids such as anthocyanins and flavan‐3‐ols are very important for the red wine quality. Anthocyanins, both free and linked to tannins, are responsible for long‐term color stability of wine (Somers, 1971), whereas tannins with different degree of polymerization are responsible for astringency and bitterness (Peleg, Gacon, Schlich, & Noble, 1999).

Volatile compounds are mainly located in the skin and in the pulp and are extracted during maceration. In addition, many volatile compounds are formed during fermentation and wine fining process. Volatile compounds of wine belong to different chemical classes including acids, alcohols, aldehydes, ketones, lactones, sulfur, and terpenes. Their concentration and relative composition discriminate different wines since they affect the aromatic character (Marti, Mestres, Sala, Busto, & Guasch, 2003; Palomo, Pérez‐Coello, Diaz‐Maroto, Viñas, & Cabezuda, 2006).

A common task of winemakers is to increase the extraction of volatile compounds, anthocyanins, and low molecular weight tannins, and limiting the extraction of seed tannins that are tough and bitter. Prefermentative cold soak (PCS), consisting in keeping the skins in the must for a few days below 10°C, seems able to meet these aims, even though controversial results have been reported in the literature (Gil‐Muñoz et al., 2009; González‐Neves, Gil, Favre, & Ferrer, 2012; Heredia et al., 2010; Puertas, Guerrero, Jurado, Jiménez, & Cantos‐Villar, 2008). This practice is currently used in the production of white wines in order to increase the extraction of volatile compounds (Peinado, Moreno, Bueno, Moreno, & Mauricio, 2004), and in red wines in order to improve the extraction of both phenols and volatiles (Gil‐Muñoz et al., 2009; Heredia et al., 2010). Some researchers reported also a positive effect of cold maceration on total anthocyanins (Gil‐Muñoz et al., 2009; Gómez‐Míguez, González‐Miret, & Heredia, 2007; Heredia et al., 2010), whereas others did not find any significant effect, or even a slight decrease (Gambacorta et al., 2011; González‐Neves et al., 2012; Puertas et al., 2008).

Aglianico, Nero di Troia, and Primitivo are nonaromatic red grape vines (*Vitis vinifera* L.) widely grown in southern Italy, from which wines with different phenolic and aromatic characteristics can be produced. The present work aimed to characterize the polyphenol and volatile fractions of wines obtained from these cultivars by application of PCS, and to assess whether this technique allows to better express their enological potential.

## 2 MATERIALS AND METHODS

### 2.1 Grapes and wine samples

The experimentation was carried out in September‐October 2015 on three batches of different red grape cultivars (Aglianico, Primitivo, and Nero di Troia), harvested from vineyards located in Southern Italy. Primitivo and Nero di Troia were from Gioia del Colle (360 m a.s.l.) and Corato (232 m a.s.l.), respectively (Puglia Region), Aglianico was from Genzano di Lucania (587 m a.s.l.) (Basilicata Region). Grapes (about 1,000 kg for each cultivar) were handpicked, packed in 20 kg perforated plastic boxes, and transported to the Pietraventosa winery (Gioia del Colle) for winemaking. The winemaking trials were conducted in 200‐L stainless steel horizontal rotary wine fermenters with a submerged cap (Industrie Fracchiolla, Adelfia, Italy). The fermenters were equipped with an insulated cooling jacket on the cylindrical body, which permits to cool quickly the de‐stemmed grapes, and microporous stainless steel bar, for oxygen distribution at the bottom of fermenter. For each cultivar, two trials (coded C and PCS) × 2 replicates were performed:

- trial C—control winemaking: Seven days of maceration at 25°C. Addition of potassium metabisulphite (20 g/100 kg); yeast (*Saccharomyces cerevisiae*, Mycoferm CRU 12, 20 g/100 kg, Everintec, Pramaggiore, Italy); yeast activator (preparation based on ammonium sulfate, diammonium phosphate, chemically inert filter and as dispersing agent, Vitamin B1, Enovit, AEB, Italy) 20 g/100 kg at the beginning of fermentation and after 3 days; O_2_, 6 mg/L/day after 2 days from the beginning of fermentation; 8 rotations per day (1 rev in 3 min), alternating clockwise and counter clockwise.
- trial PCS—prefermentative cold soak: preliminary cooling of de‐stemmed grapes at 5°C using cooling jacket, maintenance of the sample at 5°C for 48 hr, then 5 days of maceration at 25°C (7 days of maceration in total, as C).

When maceration was concluded, free‐run wine was recovered and pomace was gently pressed for obtaining press‐run wine using 43 L traditional cage staves press. The two wine fractions were blended and after 1‐week racking was done to eliminate gross lees. Wine was bottled after 6 months, without any treatments, and analyzed.

### 2.2 Chemical analyses

Representative grape samples were taken as reported in a previous paper (Gambacorta et al., 2017), and the corresponding juices were subjected to analysis of total soluble solids (TSS), pH, and titratable acidity (TA) according to EEC 2676 standard procedure (EEC, 1990). Gross composition of wine samples (ethanol, pH, TA, volatile acidity [VA], malic acid [MA], lactic acid [LA], dry reduced extract [DRE] and ashes) was assessed by WineScan (FT 120 FT‐MIR spectrometer, FOSS, Padua, Italy).

### 2.3 Polyphenols analysis

Extraction of polyphenols from grape was carried out according to Gambacorta et al. (2017). The phenolic composition of grape extracts and wines was determined by spectrophotometry as described by Di Stefano and Cravero (1991), whereas the color indices were evaluated according to the method of Glories (1984). Anthocyanin composition was determined by HPLC‐DAD according to Coletta et al. (2013), and results were expressed as mg/L of malvidin‐3‐*O*‐glucoside equivalent. Antioxidant activity (AA) was assessed using ABTS [2,2′‐azino‐bis(3‐ ethylbenzothiazoline‐6‐sulfonic acid)] assay as reported by Trani, Verrastro, Punzi, Faccia, and Gambacorta (2016), and results were expressed as μM Trolox equivalent antioxidant capacity (TEAC).

### 2.4 Volatile analysis

Volatiles were extracted by static headspace solid phase micro extraction (HS‐SPME) using a preconditioned fiber, 2 cm long 50/30 μm Divinylbenzene/Carboxen/Polydimethylsiloxane (DVB‐CAR‐PDMS) (Supelco, Bellefonte, PA). Separation of the compounds was performed using a Trace 1300 gas chromatograph (ThermoFisher Scientific, Rodano, Italy) equipped with a HP Innowax column (Agilent, Santa Clara, CA), 20 m length × 0.18 mm ID × 0.18 μm film, and coupled with an ISQ single quadrupole mass spectrometer (MS) (ThermoFisher Scientific). The detailed operative conditions have been reported in a previous paper (Trani et al., 2016). Volatiles were tentatively identified by comparing them with the linear retention index of pure standard compounds and by comparing the experimental mass spectra with those reported in the NIST Library. The identification of compound was accepted with probability higher than 80%. Volatiles were quantified using relative areas related the 2‐heptanone as internal standard.

### 2.5 Statistical analysis

All measurements were carried out in triplicate, and results were expressed as means ± *SD* (standard deviation). Statistical analysis was performed using IBM SPSS software v 19. Significant differences between control and cold macerated wines for each cultivar were determined using one‐way ANOVA and Tukey's HSD test for multiple comparisons (IBM SPSS software v 1.9). Principal component analysis (PCA) was applied to phenolic and volatile fractions in order to evaluate differences among samples using XlStat (Addinsoft, NY) software.

## 3 RESULTS AND DISCUSSION

### 3.1 Qualitative characteristics of grapes

Table 1 shows the chemical characteristics and phenolic composition of grapes. The TA and TSS values highlighted the almost satisfactory degree of ripeness reached by the grapes at the harvest time. Aglianico and Primitivo showed the highest TSS values, and Aglianico had the lowest pH and the highest TA. The total polyphenols (TP) reached the highest value in Aglianico grapes. It was confirmed by the AA value that was highest than in the other two varieties under study and showed a positive correlation with TP. Aglianico was the richest in anthocyanins, followed by Primitivo (−22%) and Nero di Troia (−37%). Regarding the qualitative differences among samples, Nero di Troia had the highest percentage of acylated anthocyanins (in particular acylated malvidin) showing a value four times highest than observed in Aglianico and Primitivo. At the same time, Aglianico had the lowest content of anthocyanins linked to *p*‐coumaric acid. These results indicate that the phenol profiles of the three cultivars under study are strongly different, confirming data reported in a previously work related to 2014 season (Gambacorta et al., 2017).

**Table 1:** Chemical characteristics and anthocyanic composition of grapes (mean values ± *SD*)

Parameters	Aglianico	Primitivo	Nero di Troia
TSS (°Brix)	21.5 ± 0.1^a^	21.2 ± 0.2^a^	20.5 ± 0.1^b^
pH	3.09 ± 0.04^b^	3.40 ± 0.05^a^	3.42 ± 0.06^a^
TA (g/L of juice)	9.52 ± 0.16^a^	6.72 ± 0.19^b^	5.43 ± 0.11^c^
TP (mg/kg)	2,383 ± 58^a^	2,100 ± 71^c^	2,268 ± 49^b^
AA (μmol/kg)	7,880 ± 442^a^	4,494 ± 263^c^	6,206 ± 150^b^
Anthocyanins (mg/kg)			
Dp	51.1 ± 4.5^a^	21.7 ± 3.4^c^	32.4 ± 3.5^b^
Cy	4.0 ± 0.9^b^	5.0 ± 1.1^ab^	5.9 ± 0.5^a^
Pt	59.5 ± 7.6^a^	33.6 ± 3.2^b^	29.7 ± 6.5^b^
Pn	35.7 ± 4.1^a^	35.4 ± 7.3^a^	16.2 ± 3.4^b^
Mv	485.1 ± 44.9^a^	336.2 ± 30.2^b^	178.2 ± 34.1^c^
Dp‐Ac	0.8 ± 0.2^b^	1.0 ± 0.1^ab^	1.3 ± 0.2^a^
Pt‐Ac	2.8 ± 0.6^b^	2.5 ± 0.5^b^	7.7 ± 1.^a^
Pn‐Ac	11.2 ± 1.1^b^	10.5 ± 2.1^b^	20.7 ± 4.2^a^
Mv‐Ac	32.2 ± 2.1^b^	23.1 ± 0.8^c^	73.8 ± 4.1^a^
*cis*‐Mv‐Cm	0.7 ± 0.1^b^	2.2 ± 0.3^a^	2.9 ± 0.6^a^
Mv‐Cf	11.2 ± 1.1^b^	12.6 ± 0.4^b^	18.0 ± 2.1^a^
Pt‐Cm	6.1 ± 0.2^b^	6.9 ± 0.3^a^	6.6 ± 0.2^ab^
Pn‐Cm	10.5 ± 0.6^c^	15.4 ± 2.1^a^	9.1 ± 0.5^b^
*trans*‐Mv‐Cm	168.0 ± 6.1^b^	178.5 ± 3.5^a^	147.6 ± 9.2^c^
Total anthocyanins	878.9 ± 51.5^a^	684.6 ± 32.2^b^	550.1 ± 45.2^c^

*Notes*

TSS, total soluble solids; TA, titratable acidity; TP, total polyphenols: as gallic acid; AA, antioxidant activity. Dp, delphinidin‐3‐*O*‐glucoside; Cy, cyanidin‐3‐*O*‐glucoside; Pt, petunidin‐3‐*O*‐glucoside; Pn, peonidin‐3‐*O*‐glucoside; Mv, malvidin‐3‐*O*‐glucoside; Dp‐Ac, delphinidin‐3‐*O*‐acetylglucoside; Pt‐Ac, petunidin‐3‐*O*‐acetylglucoside; Pn‐Ac, peonidin‐3‐*O*‐acetylglucoside; Mv‐Ac, malvidin‐3‐*O*‐acetylglucoside; *cis*‐Mv‐Cm, *cis*‐malvidin‐3‐*O*‐coumarylglucoside; Mv‐Cf, malvidin‐3‐*O*‐caffeylglucoside; Pt‐Cm, petunidin‐3‐*O*‐coumarylglucoside; Pn‐Cm, peonidin‐3‐*O*‐coumarylglucoside; *trans*‐Mv‐Cm, *trans*‐malvidin‐3‐*O*‐coumarylglucoside. In rows, data followed by different letters indicate statistically significant differences at *p *<* *0.05.

### 3.2 Chemical characteristics of wines

The chemical characteristics of wines are reported in Table 2. Cold soak had little impact on the overall basic chemical composition of the wine. It caused moderate increase of ethanol in Primitivo, and TA in Primitivo and Nero di Troia, whereas it had not any effects on Aglianico. The VA value was very low both in control and in experimental samples, because it depends greatly on grape quality and correct management of the winemaking process. It is noteworthy that malolactic fermentation was in progress in control wines and did not start in A‐PCS and P‐PCS suggesting a possible effect of cold soak on the metabolism of lactic acid bacteria.

**Table 2:** Chemical characteristics of wines (mean values ± *SD*)

Compounds	A‐C	A‐PCS	P‐C	P‐PCS	NT‐C	NT‐PCS
E (% v/v)	12.91 ± 0.12^a^	12.95 ± 0.21^a^	12.54 ± 0.11^b^	13.27 ± 0.18^a^	11.83 ± 0.09^c^	12.05 ± 0.15^c^
pH	3.16 ± 0.04^b^	3.11 ± 0.05^b^	3.50 ± 0.06^a^	3.47 ± 0.04^a^	3.60 ± 0.11^a^	3.48 ± 0.08^a^
TA (g/L)	8.85 ± 0.13^a^	8.63 ± 0.16^a^	5.81 ± 0.11^c^	6.60 ± 0.13^b^	5.39 ± 0.21^d^	5.89 ± 0.11^c^
VA (g/L)	0.30 ± 0.03	0.27 ± 0.02	0.35 ± 0.02	0.37 ± 0.03	0.30 ± 0.04	0.24 ± 0.06
MA (g/L)	4.22 ± 0.12^a^	4.15 ± 0.11^a^	1.33 ± 0.11^d^	3.23 ± 0.24^b^	2.35 ± 0.1^c^	2.38 ± 0.09^c^
LA (g/L)	0.13 ± 0.02^c^	ND	0.70 ± 0.02^a^	ND	0.25 ± 0.03^b^	0.22 ± 0.06^b^
DRE (g/L)	28.93 ± 0.16^b^	29.52 ± 0.22^a^	28.00 ± 0.11^c^	29.17 ± 0.19^ab^	26.83 ± 0.15^d^	26.25 ± 0.11^e^
Ashes (g/L)	2.27 ± 0.15^c^	2.37 ± 0.08^c^	2.92 ± 0.11^a^	3.03 ± 0.14^a^	3.09 ± 0.12^a^	2.79 ± 0.12^b^

*Notes*

A‐C, Aglianico control; A‐PCS, Aglianico prefermentative cold soak; P‐C, Primitivo control; P‐PCS, Primitivo prefermentative cold soak; NT‐C, Nero di Troia control; NT‐PCS, Nero di Troia prefermentative cold soak. E, ethanol; TA, titratable acidity: as tartaric acid; VA, volatile acidity: as acetic acid; MA, malic acid; LA, lactic acid; DRE, dry reduced extract. ND, not detected. In rows, data followed by different letters indicate statistically significant differences at *p *<* *0.05.

### 3.3 Phenolic characteristics of wines

Phenolic composition, AA, and color indices of wines are reported in Table 3. TP and proanthocyanidins (P) showed significant increase (*p *<* *0.05) in cold soak samples. In particular, the increment of TP was 5%, 18% and 14% with respect to the controls for Aglianico, Primitivo and Nero di Troia, respectively, whereas the increase in proanthocyanidins was 25%, 14%, and 7%. As a consequence, also AA was higher in the cold soak samples than in the relative controls (+13%, +15% and +6%). The higher values of P in cold soak wines led to reduction in flavans reagent with vanillin/proanthocyanidins ratio (FRV/P). This indicates that PCS applied to the investigated cultivars could favor the chromatic and tannic stabilization of wines (Suriano, Alba, Tarricone, & Di Gennaro, 2015). With regard to the color indices, color intensity (CI) significantly increased in Primitivo and Nero di Troia (+13% and +9%, respectively), whereas it did not change in Aglianico that was the most colored wine. The hue, or color tonality T, showed significant differences only in Nero di Troia samples. This parameter is computed by dividing the absorbance of wine at 420 nm with the absorbance measured at 520 nm. It increases during aging of red wine due to the reduction in anthocyanin forms (responsible for the absorbance at 520 nm) and the concomitant increase in brown pigments (accountable for the absorbance at 420 nm). The lower tonality value found in the Nero di Troia PCS sample suggests that PCS could exert an influence on the polymeric pigments production between tannins and anthocyanins. These latter seem to be preserved from precipitation, favoring color stabilization of wine. The fact that this phenomenon was observed only in Nero di Troia could be explained by the differences found in the anthocyanin composition (Table 4). From a quantitative point of view, cold soak promoted greater extraction of anthocyanins in Aglianico (+57%) and in Primitivo (+23%) and had no effects for Nero di Troia. Under the qualitative point of view, it has to be highlighted that the anthocyanin profiles of all wines were different from those of the corresponding grapes. The increase in the free form was particularly evident. Considering the increase of proanthocyanidins in all PCS samples, the decreased value of tonality and unchanged values of free forms of anthocyanins in NT‐PCS, we could infer that the polymerization process occurred with different intensity in the three wine samples. In Nero di Troia, cold soak led to an increase in the ratio between proanthocyanidins and anthocyanins, thus favoring the polymeric pigments production and determining lower value of hue than in the control. On the whole, PCS only had a quantitative impact in Aglianico and Primitivo, and never influenced the anthocyanin profile. These results confirm the controversial results reported in the literature and suggest that the effectiveness of cold soak in anthocyanin extraction could be cultivar‐dependent.

**Table 3:** Phenolic composition and color indices of wines (mean values ± *SD*)

Compounds	A‐C	A‐PCS	P‐C	P‐PCS	NT‐C	NT‐PCS
TP (mg/L)	1,943 ± 55b	2,047 ± 83ab	1,553 ± 58d	1,845 ± 48c	1,818 ± 59c	2,081 ± 88a
FRV (mg/L)	944 ± 60b	1,005 ± 37b	1,151 ± 37a	1,151 ± 39a	1,076 ± 63ab	1,006 ± 46b
P (mg/L)	1,964 ± 49c	2,454 ± 58a	1,950 ± 74c	2,221 ± 54b	2,244 ± 54b	2,407 ± 67a
FRV/P	0.48 ± 0.02b	0.41 ± 0.03b	0.59 ± 0.02a	0.52 ± 0.02b	0.48 ± 0.03b	0.42 ± 0.02c
AA (μM)	11,922 ± 296b	13,442 ± 427a	8,822 ± 260e	10,113 ± 137d	10,744 ± 189c	11,423 ± 293b
CI (pathlength 1 mm)	1.93 ± 0.03a	1.91 ± 0.02a	1.01 ± 0.03c	1.15 ± 0.05b	0.76 ± 0.03e	0.83 ± 0.02d
T (pathlength 1 mm)	0.46 ± 0.02c	0.45 ± 0.01c	0.56 ± 0.02b	0.55 ± 0.01b	0.63 ± 0.01a	0.57 ± 0.01b

*Notes*

A‐C, Aglianico control; A‐PCS, Aglianico prefermentative cold soak; P‐C, Primitivo control; P‐PCS, Primitivo prefermentative cold soak; NT‐C, Nero di Troia control; NT‐PCS, Nero di Troia prefermentative cold soak. TP, total polyphenols: as gallic acid; FRV, flavans reactive with vanillin: as (+)‐catechin; P, proanthocyanidins: as cyanidin chloride; AA, antioxidant activity; CI, color intensity; T, tonality. In rows, data followed by different letters indicate statistically significant differences at *p *<* *0.05.

**Table 4:** Anthocyanin composition of wines (mg/L, mean values ± *SD*)

Compounds	A‐C	A‐PCS	P‐C	P‐PCS	NT‐C	NT‐PCS
Dp	4.4 ± 0.1^c^	7.6 ± 0.3^a^	3.6 ± 0.2^d^	4.7 ± 0.2^bc^	5.0 ± 0.2^b^	5.4 ± 0.2^b^
Cy	0.5 ± 0.1^bc^	0.7 ± 0.1^b^	1.0 ± 0.1^a^	1.1 ± 0.1^a^	0.4 ± 0.1^c^	0.5 ± 0.1^bc^
Pt	5.7 ± 0.1^d^	9.3 ± 0.2^a^	6.2 ± 0.5^d^	8.0 ± 0.1^b^	7.5 ± 0.2^c^	7.5 ± 0.3^c^
Pn	2.6 ± 0.1^e^	4.1 ± 0.3^c^	6.3 ± 0.4^b^	8.8 ± 0.6^a^	3.1 ± 0.2^d^	2.5 ± 0.1^e^
Mv	40.0 ± 0.9^e^	68.2 ± 0.2^a^	57.1 ± 4.1^c^	65.7 ± 1.6^b^	55.8 ± 2.0^c^	55.7 ± 0.4^c^
Dp‐Ac	5.7 ± 0.2^a^	6.0 ± 0.1^a^	1.4 ± 0.1^d^	2.0 ± 0.1^b^	1.7 ± 0.1^c^	1.4 ± 0.1^d^
Pt‐Ac	0.5 ± 0.1^c^	0.6 ± 0.1^c^	0.8 ± 0.1^bc^	0.9 ± 0.1b	2.2 ± 0.2^a^	2.0 ± 0.1^a^
Pn‐Ac	ND	ND	0.3 ± 0.1^c^	0.4 ± 0.1^c^	2.0 ± 0.1^a^	1.7 ± 0.1^b^
Mv‐Ac	1.1 ± 0.1^d^	2.2 ± 0.1^c^	2.1 ± 0.1^c^	2.8 ± 0.1^c^	23.5 ± 0.1^a^	22.6 ± 0.9^a^
*cis*‐Mv‐Cm	0.8 ± 0.1^ab^	1.0 ± 0.1^a^	0.5 ± 0.1^bc^	0.5 ± 0.1^bc^	0.6 ± 0.1^b^	0.3 ± 0.1^c^
Mv‐Cf	0.5 ± 0.1^b^	0.7 ± 0.1^b^	0.7 ± 0.1^b^	0.7 ± 0.2^b^	1.4 ± 0.1^a^	1.4 ± 0.1^a^
Pt‐Cm	1.2 ± 0.1^ab^	1.4 ± 0.1^a^	1.1 ± 0.1^b^	0.9 ± 0.2^b^	1.2 ± 0.1^ab^	1.1 ± 0.1^b^
Pn‐Cm	0.3 ± 0.1^c^	0.5 ± 0.1^c^	1.2 ± 0.1^ab^	1.5 ± 0.1^a^	1.0 ± 0.1^b^	1.0 ± 0.1^b^
*trans*‐Mv‐Cm	2.5 ± 0.1^e^	4.6 ± 0.1^d^	4.5 ± 0.1^d^	6.2 ± 0.2^c^	7.6 ± 0.1^a^	7.3 ± 0.2^a^
Total anthocyanins	69.8 ± 1.5^e^	109.7 ± 2.2^ab^	86.8 ± 5.2^d^	106.5 ± 2.6^b^	113.5 ± 1.9^a^	110.4 ± 3.2^ab^

*Notes*

A‐C, Aglianico control; A‐PCS, Aglianico prefermentative cold soak; P‐C, Primitivo control; P‐PCS, Primitivo prefermentative cold soak; NT‐C, Nero di Troia control; NT‐PCS, Nero di Troia prefermentative cold soak. Dp, delphinidin‐3‐*O*‐glucoside; Cy, cyanidin‐3‐*O*‐glucoside; Pt, petunidin‐3‐*O*‐glucoside; Pn, peonidin‐3‐*O*‐glucoside; Mv, malvidin‐3‐*O*‐glucoside; Dp‐Ac, delphinidin‐3‐*O*‐acetylglucoside; Pt‐Ac, petunidin‐3‐*O*‐acetylglucoside; Pn‐Ac, peonidin‐3‐*O*‐acetylglucoside; Mv‐Ac, malvidin‐3‐*O*‐acetylglucoside; *cis*‐Mv‐Cm, *cis*‐malvidin‐3‐*O*‐coumarylglucoside; Mv‐Cf, malvidin‐3‐*O*‐caffeylglucoside; Pt‐Cm, petunidin‐3‐*O*‐coumarylglucoside; Pn‐Cm, peonidin‐3‐*O*‐coumarylglucoside; *trans*‐Mv‐Cm, *trans*‐malvidin‐3‐*O*‐coumarylglucoside. In rows, data followed by different letters indicate statistically significant differences at *p *<* *0.05. ND, not detected.

### 3.4 Volatile characteristics of wines

Table 5 shows the volatile compounds grouped into chemical classes (alcohols, esters, acids, and aldehydes). The linear retention indices, odor threshold, and odor description for each compound are reported in Table 6. The SPME/GC‐MS method allowed identification and quantification of 23 volatiles. Compounds present at a trace level (<0.005 mg/L) or identified with lower than 80% of probability have not been considered. All the identified volatile compounds derived from yeasts activity during fermentation, and thus they belong to the class of secondary aromas. It is well known that their concentration is influenced by the yeast strain, sugar content, fermentation temperature, and degree of aeration. The total content of volatile compounds ranged from 8.48 mg/L in NT‐C to 10.98 in P‐PCS. These values are about one‐half of those found in Primitivo wine in the 2013 season, and produced by using a different yeast strain (Trani et al., 2016). In comparison with control, cold soak increased the total volatiles content in all wines (+21.3%, +17.2% and +16.1% in Nero di Troia, Aglianico and Primitivo, respectively). The alcohols were the most abundant compounds in all samples, ranging from 54.2% in A‐PCS to 65.3% in NT‐C, followed by esters, ranging from 34.0% in NT‐C to 37.0% in NT‐PCS, and by acids and aldehydes with percentage lower than 0.1%. Cold soak caused slight increase in alcohols by 11.1% in Aglianico and Nero di Troia, and 6.0% in Primitivo. Alcohols are recognizable by their strong and pungent smell and taste (Kotseridis & Baumes, 2000). Among them, 1‐butanol‐3‐methyl (isoamyl alcohol), which is responsible for alcohol and fused notes, showed the highest increase. However, the concentrations found were always below the odor threshold (30 mg/L), and it should not contribute to aroma. All samples contained phenylethyl alcohol, responsible for honey, spice, rose and lilac nuances, at a concentration slightly above the odor threshold (0.75 mg/L), suggesting that it could be involved in wine aroma. Esters were the most numerous identified compounds (12), and most of them were found at concentrations above the odor threshold. Cold soak increased esters concentration by 26.0% for Aglianico, 32.2% for Primitivo and 39.9% for Nero di Troia. This finding is in agreement with results reported by Lukic′, Budic′‐Leto, Bubola, Damianijanic′, and Staver (2017) on Teran red wine. It is noteworthy that the major increase (about twice with respect to control for all three cultivars) was found for isoamyl acetate (banana odor), ethyl hexanoate (apple peel and fruit), ethyl octanoate (fruit and fat odor), and ethyl decanoate (grapey odor). Some acids and aldehydes were also identified, but at very low levels.

**Table 5:** Mean concentration and relative standard deviations (n = 6) of free volatile compounds (mg/L) of wines

Compounds	A‐C	A‐PCS	P‐C	P‐PCS	NT‐C	NT‐PCS
1‐Propanol, 2‐methyl	0.12 ± 0.01^b^	0.11 ± 0.01^b^	0.10 ± 0.01^b^	0.19 ± 0.03^a^	0.13 ± 0.01^b^	0.15 ± 0.01^ab^
1‐Butanol, 3‐methyl	3.88 ± 0.10^d^	4.49 ± 0.17^ab^	4.12 ± 0.07^c^	4.52 ± 0.24^ab^	4.33 ± 0.07^b^	4.73 ± 0.12^a^
1‐Pentanol, 3‐methyl‐	0.01 ± 0.01	0.01 ± 0.01	ND	ND	0.01 ± 0.01	ND
1‐Hexanol	0.08 ± 0.01^c^	0.06 ± 0.01^c^	0.11 ± 0.01^b^	0.06 ± 0.02^c^	0.11 ± 0.01^b^	0.18 ± 0.01^a^
1‐Hexanol, 2‐ethyl‐	0.01 ± 0.01	0.01 ± 0.01	0.02 ± 0.01	0.03 ± 0.01	0.02 ± 0.01	0.01 ± 0.01
Phenylethyl alcohol	0.94 ± 0.01^c^	0.92 ± 0.09^c^	1.36 ± 0.22^a^	1.25 ± 0.15^a^	0.94 ± 0.04^c^	1.07 ± 0.01^b^
Total alcohols	5.04 ± 0.11^c^	5.60 ± 0.10^b^	5.71 ± 0.14^b^	6.05 ± 0.16^a^	5.54 ± 0.11^b^	6.14 ± 0.14^a^
Ethyl acetate	0.65 ± 0.02^b^	0.54 ± 0.04^c^	0.55 ± 0.06^c^	0.83 ± 0.12^a^	0.57 ± 0.04^c^	0.63 ± 0.04^bc^
Ethyl isobutyrate	0.02 ± 0.01	0.02 ± 0.01	0.02 ± 0.01	0.02 ± 0.01	0.01 ± 0.01	0.01 ± 0.01
Ethyl isovalerate	0.04 ± 0.01	0.03 ± 0.01	0.02 ± 0.01	0.05 ± 0.03	0.02 ± 0.01	0.02 ± 0.01
Ethyl butyrate	0.03 ± 0.01	0.04 ± 0.01	0.04 ± 0.01	0.05 ± 0.02	0.02 ± 0.01	0.02 ± 0.01
Isoamyl acetate	0.20 ± 0.02^b^	0.46 ± 0.05^a^	0.20 ± 0.02^b^	0.46 ± 0.05^a^	0.28 ± 0.07^b^	0.49 ± 0.05^a^
Ethyl 2‐methylbutyrate	0.03 ± 0.01	0.01 ± 0.01	ND	ND	0.01 ± 0.01	0.01 ± 0.01
Ethyl hexanoate	0.49 ± 0.04^bc^	0.69 ± 0.12^a^	0.50 ± 0.01^b^	0.61 ± 0.11^a^	0.44 ± 0.03^c^	0.61 ± 0.11^a^
Ethyl heptanoate	0.01 ± 0.01	0.01 ± 0.01	ND	ND	0.01 ± 0.01	ND
Ethyl octanoate	1.57 ± 0.08^b^	2.10 ± 0.11^a^	1.65 ± 0.12^b^	2.16 ± 0.16^a^	1.20 ± 0.22^c^	1.60 ± 0.13^b^
Ethyl decanoate	0.28 ± 0.02^c^	0.37 ± 0.04^b^	0.40 ± 0.03^b^	0.48 ± 0.03^a^	0.21 ± 0.06^c^	0.36 ± 0.01^b^
Diethyl succinate	0.36 ± 0.01^a^	0.37 ± 0.02^a^	0.29 ± 0.02^b^	0.19 ± 0.06^c^	0.10 ± 0.01^d^	0.27 ± 0.01^b^
Ethyl phenylacetate	0.01 ± 0.01	0.01 ± 0.01	ND	ND	0.01 ± 0.01	0.01 ± 0.01
Total esters	3.69 ± 0.21^b^	4.65 ± 0.22^a^	3.67 ± 0.22^b^	4.85 ± 0.37^a^	2.88 ± 0.31^c^	4.03 ± 0.26^b^
Acetic acid	0.04 ± 0.01^ab^	0.03 ± 0.01^ab^	0.06 ± 0.01^a^	0.06 ± 0.02^a^	0.02 ± 0.01^b^	0.05 ± 0.01^a^
Hexanoic acid	0.01 ± 0.01	0.01 ± 0.01	ND	ND	ND	0.01 ± 0.01
Octanoic acid	0.03 ± 0.01	0.04 ± 0.01	0.02 ± 0.01	0.02 ± 0.01	0.02 ± 0.01	0.03 ± 0.01
Total acids	0.08 ± 0.01^a^	0.08 ± 0.01^a^	0.08 ± 0.02^a^	0.08 ± 0.02^a^	0.04 ± 0.01^b^	0.09 ± 0.01^a^
Nonanal	ND	ND	ND	ND	ND	0.01 ± 0.01
Furfural	0.01 ± 0.01	0.01 ± 0.01	ND	ND	0.02 ± 0.01	0.02 ± 0.01
Total aldehydes	0.01 ± 0.01	0.01 ± 0.01	ND	ND	0.02 ± 0.01	0.03 ± 0.01
Total contents	8.82 ± 0.27^b^	10.34 ± 0.25^a^	9.46 ± 0.15^b^	10.98 ± 0.48^a^	8.48 ± 0.30^c^	10.29 ± 0.40^a^

*Notes*

A‐C, Aglianico control; A‐PCS, Aglianico prefermentative cold soak; P‐C, Primitivo control; P‐PCS, Primitivo prefermentative cold soak; NT‐C, Nero di Troia control; NT‐PCS, Nero di Troia prefermentative cold soak. In rows, data followed by different letters indicate statistically significant differences at *p *<* *0.05. ND, not detected.

**Table 6:** Linear retention index, odor threshold and odor description of volatile compounds of wines

Compounds	LRI		Odor threshold (mg/L)	Odor description
	Exp.^a^	Lit.^b^		
Alcohols				
1‐Propanol, 2‐methyl	1,098	1,097	40 [1]	Wine, solvent, bitter
1‐Butanol, 3‐methyl	1,217	1,215	30 [1]	Whiskey, malt, burnt
1‐Pentanol, 3‐methyl‐	1,332	1,325	1 [2]	Herb, cacao
1‐Hexanol	1,361	1,354	8 [3]	Resin, flower, green
1‐Hexanol, 2‐ethyl‐	1,503	1,492	270 [4]	Rose, green
Phenylethyl alcohol	1,927	1,925^c^	0.75–1.1 [5]	Honey, spice, rose, lilac
Esters				
Ethyl acetate	895	893	12.3 [1]	Pineapple
Ethyl isobutyrate	965	960	0.015 [3]	Fruit, apple
Ethyl isovalerate	1,028	1,024	0.003 [3]	Fruit, lemon, anise
Ethyl butyrate	1,034	1,040	0.002 [3]	Apple
Isoamyl acetate	1,132	1,125	0.03 [1]	Banana
Ethyl 2‐methylbutyrate	1,145	1,138	0.018 [3]	Fruit, anise
Ethyl hexanoate	1,242	1,238	0.014 [3]	Apple peel, fruit
Ethyl heptanoate	1,340	1,331	0.0022 [6]	Fruit
Ethyl octanoate	1,442	1,438	0.005 [3]	Fruit, fat
Ethyl decanoate	1,653	1,647	0.2 [3]	Grape
Diethyl succinate	1,663	1,689^c^	200 [7]	Wine, fruit
Ethyl phenylacetate	1,778	1,785^c^	0.65 [5]	Fruit, sweet
Acids				
Acetic acid	1,483	1,480	20–175	Sour, pungent, vinegar
Hexanoic acid	1,855	1,847	0.42 [3]	Sweat
Octanoic acid	2,092	2,083^c^	0.5 [3]	Sweat, cheese
Aldehydes				
Nonanal	1,402	1,396	0.001 [5]	Fat, citrus, green
Furfural	1,482	1,474	14 [3]	Bread, almond, sweet

*Notes*

LRI, linear retention index. ^a^LRI on HP‐Innovax column, experimentally determined using homologous series of C8‐C30 alkanes. ^b^LRI taken from Bianchi, Careri, Mangia, and Musci (2007). ^c^LRI taken from www.flavornet.org. [1] Guth (1997); [2], Zea, Moyano, Moreno, Cortes, and Medina (2001); [3] Ferreira, Lòpez, and Cacho (2000); [4] Fazzalari, 1978; [5] Buttery, Turnbaugh, and Ling (1988); [6] Takeoka, Flath, Mon, Teranishi, and Guentert (1990); [7] Tominaga, Murat, and Dubourdieu (1998).

### 3.5 PCA analysis

**Figure 1:**
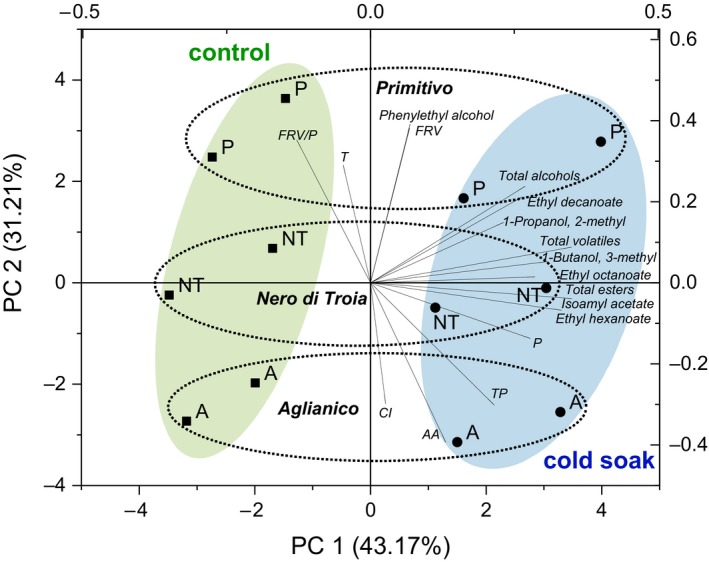
Principal component analysis: classification map of control (black square) and cold soak (black circle) wines as a function of phenolic and volatile variables. A, Aglianico; P, Primitivo; NT, Nero di Troia; AA, antioxidant activity; CI, colour intensity; FRV, flavans reactive with vanillin; FRV/P, flavans reactive with vanillin/proanthocyaninds ratio; P, proanthocyanidins; T, tonality; TP, total polyphenols

The application of PCA to phenol and volatile fractions is reported in Figure 1. In the figure, for the best of clarity, only the volatiles with the highest contribution to the total variance have been plotted. The first component (43.2% of explained variance) clearly discriminated the cold soak wines from the corresponding controls. In fact, cold soak wines were grouped on the right side and are distinguishable for their highest content in volatiles, proanthocyanidins, TP, and AA. The second component (31.2% of explained variance) discriminated the wines based on the cultivars. On this component, Primitivo wines were grouped on the top side and are distinguishable for their content in FRV, phenylethyl alcohol, tonality and RFV/P, Aglianico on the bottom side and are distinguishable for their content in AA, TP and CI, and Nero di Troia were grouped in the middle area of the plot.

## 4 CONCLUSIONS

The results of this study demonstrated that PCS applied to Aglianico, Primitivo, and Nero di Troia grapes improves the extraction of proanthocyanidins, decreasing the FRV/P ratio. As a consequence, it may be recommended for these cultivars, in order to favor wine color stabilization. This enological practice positively affected the extraction of free anthocyanins in Aglianico and Primitivo, suggesting that the results obtained were cultivar‐dependent. Concerning the volatile compounds, the treatment increased extraction of esters that potentially contribute to the enrichment of fruity odors of wines.

## ETHICAL STATEMENT AND CONFLICT OF INTEREST

I testify on behalf of all coauthors that the research in this article did not involve human experimentation and/or animal testing. All authors have read, approved and are fully conversant with the manuscript; all authors were also aware of its submission to *Food Science & Nutrition* and declared no conflict of interest.
